# Memantine-Induced Cholestasis and Acute Hepatitis in an 8-Week-Old Term Infant

**DOI:** 10.14309/crj.0000000000002301

**Published:** 2026-09-18

**Authors:** Briona Thompson, Kadakkal Radhakrishnan

**Affiliations:** 1 Department of Gastroenterology, Cleveland Clinic Foundation, Cleveland, OH

**Keywords:** memantine, cholestasis, breast-feeding

## Abstract

Cholestasis and hepatitis in infants occur for various reasons and require further evaluation. We present an 8-week-old term male infant with cholestasis secondary to breast milk transference of memantine. To the best of our knowledge, this is the first case associated with cholestasis and acute hepatitis with memantine transference through breast-feeding.

## INTRODUCTION

Work-up for neonatal cholestasis in infants has a broad differential, including drug-induced liver injury. We present a case of a 2-month-old patient with direct hyperbilirubinemia, jaundice, and weight loss, concerning for cholestasis and acute hepatitis

## CASE REPORT

A 2-month-old ex-full-term male infant presented with growth faltering and scleral icterus. The infant did not have newborn jaundice at birth and developed jaundice in the past 2 weeks. The mother had negative prenatal laboratory test results with negative HBsAg and hepatitis C, nonreactive for HIV. On admission, laboratory values were significant for elevated liver function tests (Table 1, day 0). International normalized ratio was mildly elevated at 1.5, and newborn screen was unremarkable. Physical examination was significant for jaundice, small flat hemangioma on the left lateral abdomen, and liver <1 cm below the right costal margin in the midclavicular line. Since birth, the patient had dropped greater than 2 SDs of *Z* score for weight. Stools were observed to be yellow in color.

**Table 1.** Laboratory levels of the patient since initial abnormal laboratory test results

Number of days since initial laboratory test results	Alanine aminotransferase (U/L)	Aspartate aminotransferase (U/L)	Total bilirubin (mg/dL)	Direct bilirubin (mg/dL)
0	302	202	6.5	3.8
5	255	172	4.6	3.6
36	220	120	0.5	0.3
106	41	48	<0.2	<0.1
168	41	56	<0.2	0.1

The right upper quadrant ultrasound was unremarkable. Initial differentials included biliary atresia or other causes of neonatal cholestasis, including alpha-1 antitrypsin and Alagille syndrome. Gamma-glutamyl transferase (GGT) was elevated at 433 U/L, excluding differentials, including progressive familial intrahepatic cholestasis, and bile acid synthetic disorders. Since the newborn screen was unremarkable, the chance of cystic fibrosis, galactosemia, and other inherited disorders of metabolic syndrome were less likely in possibility. Cytomegalovirus deoxyribonucleic acid was negative in urine. Fat-soluble vitamins, including vitamins A and E, were unremarkable, except for low vitamin D 25-hydroxy (11 ng/mL). Protein induced in vitamin K absence-II was elevated to 134.2 ng/mL suggesting vitamin K deficiency. Echocardiogram and head ultrasound were unremarkable along with normal x-ray of the skull and spine with no butterfly vertebrae identified.

Mother was taking memantine for severe refractory migraines at the time and throughout her pregnancy as part of a clinical trial, reporting significant benefits. Mother was not taking any other medications other than prenatal vitamins. Baby was being exclusively breast-fed. Patient was started on ursodeoxycholic acid and switched to formula high in medium-chain triglycerides (Progestimil) to potentially eliminate memantine-induced cholestasis excreted in breast milk. The patient was discharged from the hospital without a liver biopsy due to significant improvement in jaundice after being placed on formula and off breast milk. On the day of discharge/day 5, liver function tests were still elevated, with an improvement of direct hyperbilirubinemia since admission (Table 1). Matrix Metalloproteinase 7 of 18.1 mg/mL (indeterminate for biliary atresia). Cholestasis genetic panel by prevention was indeterminate for being heterozygous in the SERPINA1 gene, a gene involved in alpha 1 antitrypsin deficiency—an autosomal recessive disorder.

There continued to be clinical and laboratory improvement on ursodeoxycholic acid and continued formula change with direct hyperbilirubinemia and liver function tests down-trending and most normalizing within 100 days post-discharge (Table 1). Given that the previous laboratory test results were either unremarkable or indeterminate for a diagnosis, maternal medications were further explored. Mother reintroduced breast milk, and memantine levels were tested in the serum of the patient and mother. Memantine serum levels were 93.76 ng/mL in the mother and 1.135 ng/mL in the infant. The patient had a higher milk/plasma ratio of 1.42. Given these findings, the elevated liver enzymes and cholestasis were most likely secondary to memantine transference in the mother's breast milk (Table 2).

**Table 2.** Summary of cholestasis work-up during hospital admission

Test	Result
INR	1.5 (0.9–1.3)
GGT	433 U/L (10–70 U/L)
Cytomegalovirus qualitative nonplasma PCR	Negative
Vitamin A (retinol)	0.20 mg/L (0.20–0.50 mg/L)
Vitamin D 25 hydroxy	11 ng/mL (31–80 ng/mL)
Vitamin E (alpha-tocopherol)	2.2 mg/L (2–6 mg/L)
Protein induced in vitamin K absence-II	134.2 ng/mL (0–7.4 ng/mL)
Matrix metalloproteinase 7	18.1 mg/mL (3.6–10.5 mg/mL)
Cholestasis genetic panel by prevention	Indeterminate for heterozygosity of SERPINA1 gene

Normal ranges of laboratory values in parenthesis. CYP, cytochrome P450; GGT, gamma-glutamyl transferase; INR, international normalized ratio; PCR, polymerase chain reaction.

## DISCUSSION

Memantine is considered a pregnancy category B drug with insufficient data in pregnant patients. The excretion of memantine in human milk is unknown.^1^ Memantine is an N-methyl-D-aspartate receptor antagonist, minimally metabolized by the liver and excreted mainly in the urine. There are only 2 other case reports that describe memantine-induced hepatitis with cholestasis in 2 different elderly patients. These patients' laboratory test results and symptoms improved within 3 weeks of eliminating memantine in the patient's diet. None of these case reports were associated with pediatric cholestasis or breast-feeding.^2,3^ In our case, the patient did have detectable memantine serum levels that were transmitted through breast milk. There was also a high milk/plasma ratio. Given the lack of insufficient data maternal use of memantine with maternal excretion in breast milk, other diagnosis had to be fully excluded to consider this diagnosis.

Although the patient was heterozygous for SERPINA1 gene, the possibility of alpha 1 antitrypsin deficiency was unlikely. A liver biopsy was strongly considered but given the clinical examination (lack of significant hepatomegaly), improvement in laboratory test results, Matrix Metalloproteinase 7 being indeterminate; the possibility of biliary atresia was less likely. Protein induced in vitamin K absence-II demonstrated vitamin K deficiency which is common in cholestatic liver disease.^4^ The normal cholestasis gene panel and the newborn screens made other genetic etiologies including progressive familial intrahepatic cholestasis, Alagille syndrome, cystic fibrosis, galactosemia, and other metabolic disorders unlikely.

Drug-induced liver injury should always be considered in cholestasis and acute hepatitis, but it is a diagnosis of exclusion. This clinical scenario was unique in that the source of medication was through maternal breast milk. Many other medications have been described as causing a variety of drug-induced liver disorders, including cholestasis, often due to alteration in drug metabolism including cytochrome P450 (CYP) polymorphisms.^5^ Memantine has a minimal role with CYP with some studies showing in vitro selective inhibition of CYP2B6 activity at enough blood concentration.^6^ This is the first report of memantine-induced cholestasis in a neonate/pediatric patient. This highlights the importance of taking a closer look at maternal medications and pediatric medications when evaluating acute hepatitis and cholestasis in neonates and children, in addition to the other work-up.

## DISCLOSURES

Author contributions: B. Thompson: writing-original draft, review and editing. K. Radhakrishnan: review and editing of the manuscript, supervising and reviewing final draft. B. Thompson is the article guarantor.

Financial disclosure: None to report.

Informed consent was obtained for this case report.

## ABBREVIATIONS:

GGT: gamma-glutamyl transferase
HBsAg: hepatitis B surface antigen
